# Dietary fatty acids intake and the risk of advanced cardiovascular-kidney-metabolic syndrome: evidence from NHANES 1999–2018

**DOI:** 10.1007/s10238-026-02251-y

**Published:** 2026-07-17

**Authors:** Qiuhui Wang, Fengming Liang, Hongyang Xu, Yang Chen

**Affiliations:** 1 Department of Critical Care Medicine, The Affiliated Wuxi People’s Hospital of Nanjing Medical University, Wuxi People’s Hospital, Wuxi Medical Center, Nanjing Medical University, No. 299, Qingyang Road, Wuxi, Jiangsu 214023 China; 2 Department of Cardiovascular and Metabolic Medicine, Institute of Life Course and Medical Sciences, University of Liverpool, Liverpool, UK; 3 Liverpool Centre for Cardiovascular Science at University of Liverpool, Liverpool John Moores University and Liverpool Heart and Chest Hospital, William Henry Duncan Building, 6 West Derby Street, Liverpool, L7 8TX UK

**Keywords:** Dietary fatty acids, Cardiovascular-kidney-metabolic syndrome, Saturated fatty acids, Monounsaturated fatty acids, Polyunsaturated fatty acids, NHANES

## Abstract

Cardiovascular-Kidney-Metabolic syndrome (CKM) is a multisystem disorder involving cardiovascular, renal, and metabolic dysfunction, with advanced stages (3–4) linked to high morbidity and mortality. Dietary fatty acids (FA) play critical roles in lipid metabolism, insulin sensitivity, and inflammation, yet their associations with CKM progression remain unclear. Data were from National Health and Nutrition Examination Survey, with stages 3–4 defined as advanced CKM. Dietary intake of total fat acids (TFA), saturated fatty acids (SFA), monounsaturated fatty acids (MUFA), and polyunsaturated fatty acids (PUFA) was derived from two 24-h recalls and energy-adjusted using the residual method. Multivariable logistic regression and restricted cubic splines assessed associations between FA and advanced CKM. Sensitivity and subgroup analyses were also performed. Among 27,166 participants (median age: 55.0 years; 52.2% male), 6,634 were classified in advanced CKM stages (3–4). Multivariable logistic regression showed that higher intakes of TFA, SFA, and MUFA were significantly associated with advanced CKM. Compared with the lowest quartile, adjusted odds ratios in the highest quartile were 1.35 (95% CI: 1.05–1.40), 1.26 (95% CI: 1.09–1.37), 1.30 (95% CI: 1.10–1.55), and 1.26 (95% CI: 1.09–1.47), respectively. Restricted cubic spline analyses supported positive linear associations across all four fatty acid categories. Findings remained robust in sensitivity analyses excluding cancer patients, and consistent across key subgroups, with no significant interactions observed. Higher dietary intakes of TFA, SFA, MUFA, and PUFA were associated with greater odds of advanced CKM stages. These findings suggest that dietary fat quantity and composition may be important correlates of CKM severity and warrant further investigation in prospective studies.

**Supplementary Information:**

The online version contains supplementary material available at https://doi.org/10.1007/s10238-026-02251-y.

## Introduction

Interactions among cardiovascular diseases (CVD), chronic kidney disease (CKD), and metabolic disorders give rise to Cardiovascular-Kidney-Metabolic syndrome (CKM), a cross-disease pathology in which individuals often progress to more advanced stages [1]. The American Heart Association (AHA) recently introduced a five-stage CKM classification framework, ranging from stage 0 (absence of risk factors) to stage 4 (clinical CVD) [2]. Approximately 15% of individuals are classified as having advanced CKM (stages 3–4), which is associated with substantially increased cardiovascular, renal, and metabolic risk [3, 4].

Dietary and nutritional factors play a critical role in the onset and progression of CKM, with FA intake being particularly significant [5]. Fatty acids (FAs) can be categorized into SFAs, MUFAs, and PUFAs [6]. Through modulating lipid metabolism, inflammatory responses, and insulin sensitivity, these FAs are directly linked to cardiovascular and renal health [7, 8].

Excess adiposity, insulin resistance, oxidative stress, and chronic inflammation are key mechanisms underlying CKM development, contributing to the progression of metabolic dysfunction, kidney disease, and cardiovascular risk [9–11]. Dietary FAs may influence these processes through their effects on lipid metabolism, inflammation, and glucose homeostasis [12].

However, the specific mechanisms by which different types of FAs contribute to the progression of CKM remain incompletely understood and require further investigation. A large body of randomized controlled trials and prospective cohort studies has shown that dietary fat acids (FA) intake is closely associated with various chronic diseases [13], including obesity [14], diabetes [15], CVD [16], and cancer [17]. Saturated fatty acids (SFA) are generally linked to adverse health outcomes, whereas polyunsaturated fatty acids (PUFA) may exert protective effects [18]. At the same time, substantial evidence indicates that unsaturated FA play a beneficial role in improving lipid profiles, alleviating inflammatory responses, and regulating metabolic processes [19]. These findings suggest that the intake of different FA types may differentially influence the likelihood of advanced CKM stages.

Therefore, this study aimed to assess the relationship between dietary FA intake and the likelihood of advanced CKM stages (3 and 4), based on data derived from the National Health and Nutrition Examination Survey (NHANES).

## Methods

### Data sources

The present study utilized data from NHANES, a nationwide survey carried out by the Centers for Disease Control and Prevention from 1999 to 2018. NHANES is intended to provide a representative picture of the health and nutrition of the civilian, non-institutionalized U.S. population. It employs a stratified, multistage probability sampling approach and combines standardized interviews with comprehensive clinical assessments. Ethical approval was granted by the National Center for Health Statistics Institutional Review Board, and informed consent was obtained from all participants. Compliance with STROBE guidelines was ensured (shown in Supplementary Table S1).

### Participants and study outcome

**Fig. 1:**
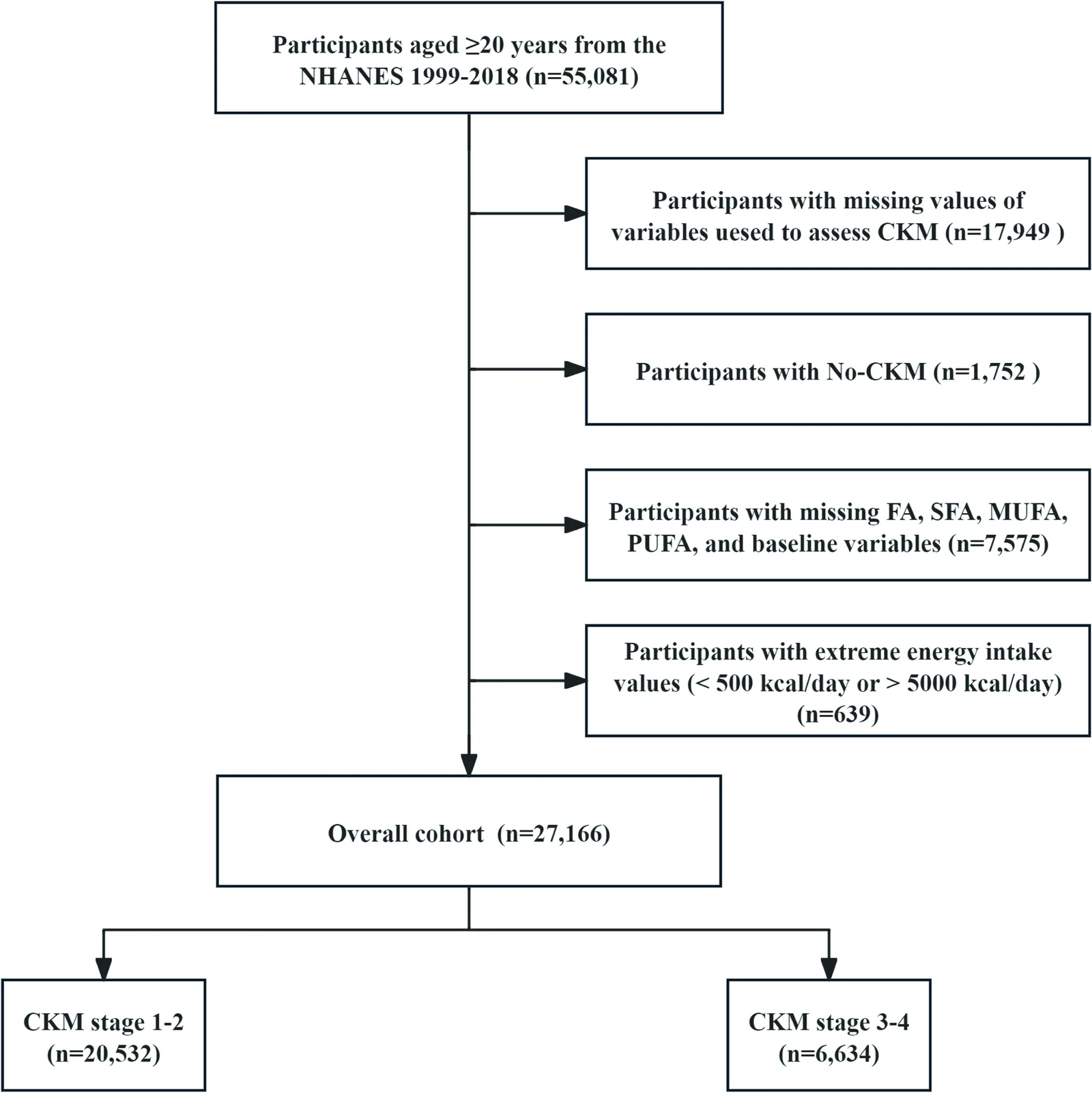
Screening flow of participants. NHANES, National Health and Nutrition Examination Survey; CKM, cardiovascular-kidney-metabolic syndrome; FA, fatty acids; SFA, saturated fatty acids; MUFA, monounsaturated fatty acids; PUFA, polyunsaturated fatty acids

The study population selection process is illustrated in Fig. 1, with the following exclusion criteria applied: (1) 17,949 participants with missing values of variables used to assess CKM; (2) 1,752 without CKM; (3) 7,575 with missing dietary information of total fatty acids (TFA), SFA, monounsaturated fatty acids (MUFA), PUFA, and baseline variables; and (4) 639 with implausible energy intake values, defined as less than 500 kcal/day or greater than 5000 kcal/day. Study outcome was the presence of advanced CKM (stages 3–4), compared with lower stages (1–2).

### Extracted covariates

The covariates encompassed a range of factors, including age, sex (female or male), body mass index [BMI] (was calculated as weight in kilograms divided by height in metres squared), race and ethnicity (Mexican American, non-Hispanic Black, non-Hispanic White, Hispanic, and others), poverty income ratio, education level, marital status, Alcohol status, and smoking status, physical activity, hypertension, diabetes, cardiovascular diseases, and biomarkers (total cholesterol, high-density lipoprotein cholesterol [HDL-C], estimated glucose disposal rate [eGFR]; urinary albumin to creatinine ratio [UACR].

### Dietary fatty acid assessment

Each participant completed two non-consecutive 24-hour dietary recalls, administered by trained interviewers. To minimise bias and enhance recall accuracy, the interviews employed the United States Department of Agriculture’s validated five-step Automated Multiple-Pass Method. Reported foods and beverages were subsequently matched with nutrient information from the USDA Food and Nutrient Database for Dietary Studies, which contains detailed FA composition for each item [20]. For each individual, daily consumption of TFA, SFA, MUFA, and PUFA was calculated by averaging the values from two separate 24-hour dietary recalls and expressed in grams per day (g/day).

To minimise confounding by total energy intake, energy adjustment was performed using the residual method, a widely accepted approach in nutritional epidemiology. Specifically, separate linear regression models were fitted for TFA, SFA, MUFA, and PUFA, with total energy intake as the independent variable. The residuals from these models were standardized and used as energy-adjusted measures of FA intake [21, 22]. Participants were subsequently categorized into quartiles according to the distribution of these standardized residual values for logistic regression analyses. In addition, restricted cubic spline analyses were performed using the standardized residual values as continuous exposure variables to investigate potential dose–response relationships. Therefore, all association analyses were based on energy-adjusted FA intake rather than absolute dietary intake.

### Definitions of CKM and stages

CKM was staged from 0 to 4 according to AHA criteria, incorporating measures of adiposity, glycaemic status, blood pressure, lipid profile, kidney disease stage, predicted 10-year CVD risk (AHA PREVENT equations), and presence of clinical CVD [23–28]. Stages 1–2 represent early metabolic or renal risk, while stages 3–4 indicate advanced disease characterized by very high CKD risk, elevated CVD risk, or established CVD. Detailed operational definitions and adapted thresholds for NHANES are provided in Supplementary Tables S2–S4.

### Statistical analysis

This study did not contain any missing data. In addition to being classified into four categories according to CKM stage (1–4), patients with CKM were also grouped into quartiles (Q1–Q4) based on their dietary FA intake. Given the complex multistage sampling design of the NHANES, all analyses incorporated sampling weights, strata, and primary sampling units to obtain nationally representative estimates. For pooled analyses across survey cycles, NHANES examination weights were recalculated according to National Center for Health Statistics recommendations. Continuous variables were presented as weighted means with standard errors, whereas categorical variables were expressed as weighted percentages. Group differences were evaluated using design-based Kruskal–Wallis tests for continuous variables and Rao–Scott adjusted Chi-squared tests for categorical variables.

Restricted cubic spline (RCS) analyses were performed to explore potential nonlinear associations between dietary FA intake and advanced CKM syndrome. RCS curves were fitted using weighted logistic regression models with normalized sampling weights and were adjusted for age, sex, marital status, education level, race/ethnicity, poverty income ratio, physical activity, carbohydrate intake, protein intake, and total energy intake. Overall and nonlinear associations were assessed using Wald tests.

Associations between FA intake quartiles and advanced CKM syndrome (Stages 3–4) were examined using survey weighted logistic regression models. Two models were constructed: Model 1 was unadjusted; Model 2 was adjusted for age, sex, marital status, education level, race/ethnicity, poverty income ratio, physical activity, carbohydrate intake, protein intake, and total energy intake. Results were reported as odds ratios (ORs) with corresponding 95% confidence intervals (CIs).

Sensitivity analyses were conducted after excluding participants with a history of cancer to minimise the potential influence of cancer-related changes in nutritional status and dietary intake. Prespecified subgroup analyses were further performed according to age (< 65 vs. ≥65 years), sex, and body mass index (BMI; <30 vs. ≥30 kg/m²). Interaction effects were evaluated by including multiplicative interaction terms in survey weighted logistic regression models, and corresponding P values for interaction were reported.

All statistical procedures were performed using R software (version 4.4.2, R Foundation for Statistical Computing, Vienna, Austria). A two-tailed probability value below 0.05 was considered to indicate statistical significance.

## Results

### Baseline characteristics

Table 1 presents the baseline characteristics of all participants, stratified by CKM stages. A total of 27,166 eligible participants were included in this study, representing a weighted population of 113,126,949 US adults (weighted population: 92,895,426 individuals with CKM stages 1–2 and 20,231,523 individuals with CKM stages 3–4). The mean age was 50.9 ± 16.2 years, and 52.2% were male. Participants were categorized according to CKM stage: 20,532 individuals were in stages 1–2 (mean age: 47.2 ± 14.2 years; 50.0% male), while 6,634 individuals were in stages 3–4 (mean age: 69.0 ± 12.2 years; 59.1% male).

**Table 1:** Baseline characteristics categorized by advanced and early CKM stages

Characteristics	Total (*n* = 27166)	CKM stages 1–2 (*n* = 20532)	CKM stages 3–4 (*n* = 6634)	*P*
Weighed number	113,126,949	92,895,426	20,231,523	
Age, years	50.9 ± 16.2	47.2 ± 14.2	69.0 ± 12.2	< 0.001
Male, n (%)	14,193 (52.2)	10,270 (50)	3923 (59.1)	< 0.001
Race and ethnicity, n (%)				< 0.001
Non-Hispanic White	12,753 (46.9)	8789 (42.8)	3964 (59.8)	
Non-Hispanic Black	5581 (20.5)	4368 (21.3)	1213 (18.3)	
Mexican American	4539 (16.7)	3765 (18.3)	774 (11.7)	
Hispanic and Others	4293 (15.8)	3610 (17.6)	683 (10.3)	
Body mass index, kg/m^2^	29.8 ± 6.4	29.8 ± 6.4	29.8 ± 6.1	0.718
Waist measurement. cm	101.9 ± 15.4	101.2 ± 15.4	105.1 ± 14.8	< 0.001
Comorbidities, n (%)				
Diabetes	6177 (22.7)	3380 (16.5)	2797 (42.2)	< 0.001
Cardiovascular diseases	3543 (10.5)	0 (0.0)	3543 (58.6)	< 0.001
Hypertensive	19,917 (73.3)	14,173 (69)	5744 (86.6)	< 0.001
Cancer	3015 (11.1)	1494 (7.3)	1521 (22.9)	< 0.001
Poverty income ratio, n (%)				< 0.001
< 1.0	5106 (18.8)	3856 (18.8)	1250 (18.8)	
1.0–3.0	11,741 (43.2)	8298 (40.4)	3443 (51.9)	
> 3.0	10,319 (38.0)	8378 (40.8)	1941 (29.3)	
Education, n (%)				< 0.001
Less than high school	3244 (11.9)	2072 (10.1)	1172 (17.7)	
High school or equivalent	10,421 (38.4)	7645 (37.2)	2776 (41.8)	
College or above	13,501 (49.7)	10,815 (52.7)	2686 (40.5)	
Marital status, n (%)				< 0.001
Unmarried	3579 (13.2)	3253 (15.8)	326 (4.9)	
Married	16,841 (62.0)	13,030 (63.5)	3811 (57.4)	
Divorcee	6746 (24.8)	4249 (20.7)	2497 (37.6)	
Smoking status, n (%)				< 0.001
Never smoker	14,054 (51.7)	11,195 (54.5)	2859 (43.1)	
Former smoker	7743 (28.5)	4885 (23.8)	2858 (43.1)	
Current smoker	5369 (19.8)	4452 (21.7)	917 (13.8)	
Alcohol consumption, n (%)				< 0.001
Non-drinker	17,224 (63.4)	11,850 (57.7)	5374 (81)	
Mild to moderate	6236 (23.0)	5352 (26.1)	884 (13.3)	
Heavy	3706 (13.6)	3330 (16.2)	376 (5.7)	
Physical activity, n (%)				< 0.001
Less than moderate	15,921 (58.6)	11,637 (56.7)	4284 (64.6)	
Moderate	6763 (24.9)	5028 (24.5)	1735 (26.2)	
Vigorous	4482 (16.5)	3867 (18.8)	615 (9.3)	
Laboratory indicators				
Total cholesterol, mg/dL	199.8 ± 41.9	202.4 ± 41.1	187.8 ± 43.5	< 0.001
HDL-C, mg/dL	52.4 ± 16.1	52.8 ± 16.2	50.5 ± 15.5	< 0.001
eGFR, ml/min/1.73m^2^	90.5 ± 21.6	95.0 ± 18.7	69.7 ± 21.8	< 0.001
UACR, mg/g	27.3 ± 95.4	19.6 ± 68.9	62.4 ± 166.1	< 0.001
Energy, kcal/day	2139.1 ± 869.6	2200.3 ± 882.3	1858.1 ± 746.8	< 0.001
Protein, g/day	81.8 ± 37.4	84.0 ± 38.0	71.9 ± 32.9	< 0.001
Carbohydrate, g/day	252.6 ± 111.5	258.7 ± 113.7	225.0 ± 95.9	< 0.001
TFA, g/day	80.6 ± 43.1	80.4 ± 43.9	81.6 ± 39.3	< 0.001
SFA, g/day	26.1 ± 15.5	26.1 ± 15.8	26.2 ± 14.2	0.005
MUFA, g/day	29.3 ± 16.7	29.2 ± 16.9	29.8 ± 15.5	< 0.001
PUFA, g/day	18.0 ± 11.6	17.9 ± 11.7	18.3 ± 11.0	< 0.001

Data are presented as weighted mean ± standard deviation for continuous variables and unweighted n (weighted %) for categorical variables. P values were calculated using design based Kruskal–Wallis tests for continuous variables and Rao–Scott adjusted Pearson Chi² tests for categorical variables, accounting for the complex sampling design of NHANES, including sampling weights, strata, and primary sampling units

Patients in the advanced CKM stages (3–4) were older, had a higher proportion of males, and exhibited a significantly greater prevalence of comorbid diabetes, hypertension, and cardiovascular disease. Compared with those in the early stages, individuals in the advanced stages had lower levels of education and income, were more likely to have insufficient physical activity, but showed lower rates of smoking and alcohol consumption.

**Fig. 2:**
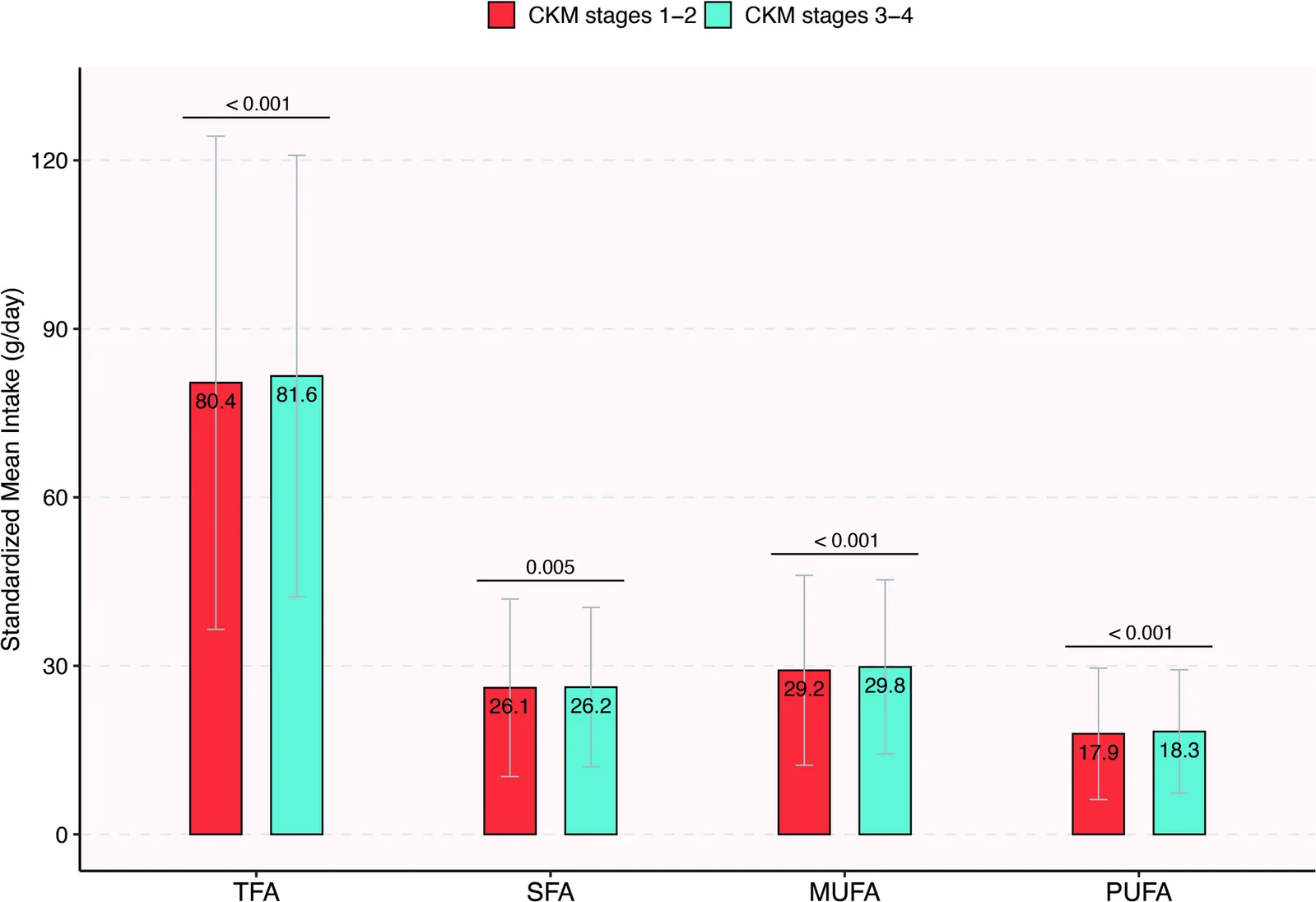
Distribution of standardized dietary fatty acid intake among participants with advanced and early CKM stages. Data are presented as weighted means ± weighted SDs of standardized dietary fatty acid intake. Error bars indicate weighted SDs. Differences between CKM stages 1–2 and CKM stages 3–4 were assessed using design based Kruskal–Wallis tests accounting for NHANES sampling weights, strata, and primary sampling units. P values are shown above each pair of bars. CKM, cardiovascular-kidney-metabolic syndrome; MUFA, monounsaturated fatty acids; PUFA, polyunsaturated fatty acids; SD, standard deviation; SFA, saturated fatty acids; TFA, total fatty acids

Figure 2 illustrates the standardized residual intake of FA across different CKM stages. Compared with patients in stages 1–2, those in stages 3–4 showed slightly higher intakes of TFA (81.6 vs. 80.4 g/day, *P* < 0.001), SFA (26.2 vs. 26.1 g/day, *P* = 0.005), MUFA (29.8 vs. 29.2 g/day, *P* < 0.001), and PUFA (18.3 vs. 17.9 g/day, *P* < 0.001).

### Associations of fatty acid intake with advanced CKM

**Fig. 3:**
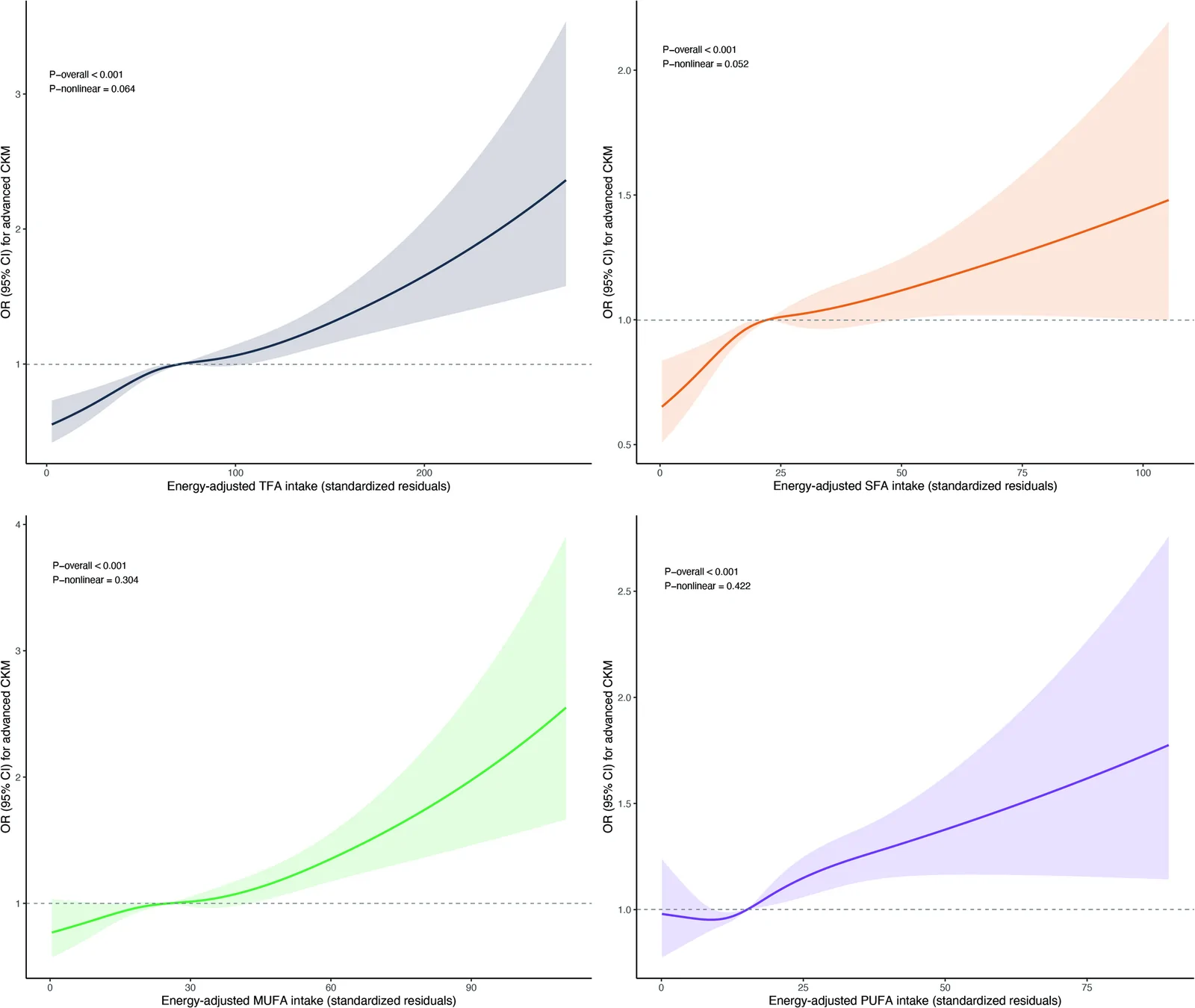
Restricted cubic spline analyses of the associations between energy-adjusted dietary fatty acid intake and advanced stages of cardiovascular-kidney-metabolic syndrome. Solid lines represent adjusted odds ratios (ORs), and shaded bands indicate 95% confidence intervals (CIs). The x-axis represents energy-adjusted fatty acid intake expressed as standardised residual values derived using the residual method rather than absolute intake (g/day). The median residual value was used as the reference. Histograms show the distribution of energy-adjusted fatty acid intake. P-overall represents the overall association, whereas P-nonlinear evaluates departure from linearity. CI, confidence interval; MUFA, monounsaturated fatty acids; OR, odds ratio; PUFA, polyunsaturated fatty acids; SFA, saturated fatty acids; TFA, total fatty acids

Figure 3 presents the RCS analyses based on weighted logistic regression models incorporating normalized NHANES sampling weights. Increasing intakes of TFA, SFA, MUFA, and PUFA were all associated with progressively higher odds of advanced CKM stages (all P for overall < 0.05). The dose-response relationships appeared approximately linear throughout the observed intake ranges, with no significant evidence of nonlinearity for any FA (all P for nonlinearity > 0.05).

The multivariable logistic regression analysis demonstrated that higher quartiles of dietary FA intake were significantly associated with advanced CKM stages (Table 2). For TFA, compared with participants in Q1, those in Q2, Q3, and Q4 had significantly higher odds of advanced CKM stages, with adjusted ORs of 1.22 (95% CI: 1.08–1.39, *P* = 0.002), 1.36 (95% CI: 1.19–1.57, *P* < 0.001), and 1.35 (95% CI: 1.05–1.40, *P* < 0.001), respectively. Similarly, higher SFA intake was associated with increased odds of advanced CKM stages across all higher quartiles, with adjusted ORs of 1.15 (95% CI: 1.02–1.30, *P* = 0.023), 1.18 (95% CI: 1.03–1.36, *P* = 0.017), and 1.26 (95% CI: 1.09–1.37, *P* = 0.002) for Q2, Q3, and Q4, respectively. For MUFA, significant associations were observed in Q3 (OR: 1.30, 95% CI: 1.12–1.50, *P* < 0.001) and Q4 (OR: 1.30, 95% CI: 1.10–1.55, *P* = 0.003), whereas the association for Q2 did not reach statistical significance (*P* = 0.056). For PUFA, participants in Q3 and Q4 exhibited significantly higher odds of advanced CKM stages, with adjusted ORs of 1.16 (95% CI: 1.01–1.33, *P* = 0.037) and 1.26 (95% CI: 1.09–1.47, *P* = 0.003), respectively.

**Table 2:** The associations between dietary fatty acid intake quartiles and advanced cardiovascular-kidney-metabolic syndrome

	Model 1		Model 2	
	OR (95% CI)	*P*	OR (95% CI)	*P*
**TFA quartiles**				
Q1: 35.2 (26.5–41.7) g/day	*Reference*		*Reference*	
Q2: 59.1 (53.6–64.9) g/day	1.51 (1.36, 1.68)	< 0.001	1.22 (1.08, 1.39)	0.002
Q3: 84.0 (77.3–91.9) g/day	1.58 (1.43, 1.76)	< 0.001	1.36 (1.19, 1.57)	< 0.001
Q4: 125.8 (111.1–148.7) g/day	1.24 (1.10, 1.39)	< 0.001	1.35 (1.05, 1.40)	< 0.001
**SFA quartiles**				
Q1: 10.2 (7.5–12.4) g/day	*Reference*		*Reference*	
Q2: 18.2 (16.3–20.2) g/day	1.39 (1.25, 1.54)	< 0.001	1.15 (1.02, 1.30)	0.023
Q3: 26.9 (24.4–29.6) g/day	1.38 (1.25, 1.53)	< 0.001	1.18 (1.03, 1.36)	0.017
Q4: 41.9 (36.9–50.5) g/day	1.19 (1.07, 1.32)	0.001	1.26 (1.09, 1.37)	0.002
**MUFA quartiles**				
Q1: 11.9 (8.9–14.5) g/day	*Reference*		*Reference*	
Q2: 21.0 (18.8–23.1) g/day	1.42 (1.26, 1.59)	< 0.001	1.16 (1.00, 1.34)	0.056
Q3: 30.4 (27.7–33.3) g/day	1.49 (1.34, 1.65)	< 0.001	1.30 (1.12, 1.50)	< 0.001
Q4: 46.3 (40.7–55.7) g/day	1.22 (1.09, 1.37)	< 0.001	1.30 (1.10, 1.55)	0.003
**PUFA quartiles**				
Q1: 6.6 (4.8–8.1) g/day	*Reference*		Reference	
Q2: 12.3 (10.9–13.6) g/day	1.28 (1.15, 1.43)	< 0.001	1.05 (0.92, 1.20)	0.436
Q3: 18.4 (16.6–20.3) g/day	1.41 (1.26, 1.57)	< 0.001	1.16 (1.01, 1.33)	0.037
Q4: 29.5 (25.5–36.3) g/day	1.23 (1.10, 1.38)	< 0.001	1.26 (1.09, 1.47)	0.003

Model 1: Unadjusted;

Model 2: Adjusted for age, sex, marital status, education, race, poverty income ratio, physical activity, carbohydrate intake, TFA、protein, and energy intake

ORs and 95% CIs were estimated using survey weighted logistic regression models accounting for NHANES sampling weights, strata, and primary sampling units. Participants in the lowest quartile (Q1) of dietary fatty acid intake were used as the reference group

Abbreviations: CI, confidence interval; MUFA, monounsaturated fatty acids; OR, odds ratio; PUFA, polyunsaturated fatty acids; SFA, saturated fatty acids; TFA, total fatty acids

### Sensitivity analyses

In the sensitivity analysis excluding CKM patients with a history of cancer (Supplementary Table S5), the associations between FA intake and advanced CKM stages remained largely consistent with the main findings. For TFA, participants in Q2–Q4 had significantly higher odds of advanced CKM stages compared with those in Q1, with adjusted ORs of 1.20 (95% CI: 1.04–1.38, *P* = 0.010), 1.33 (95% CI: 1.14–1.54, *P* < 0.001), and 1.28 (95% CI: 1.05–1.56, *P* = 0.016), respectively. For SFA, significant associations were observed in Q3 (OR: 1.20, 95% CI: 1.03–1.40, *P* = 0.021) and Q4 (OR: 1.20, 95% CI: 1.01–1.44, *P* = 0.039), whereas the association for Q2 was not statistically significant (*P* = 0.076). For MUFA, elevated odds of advanced CKM stages were observed across Q2–Q4, with adjusted ORs of 1.19 (95% CI: 1.02–1.40, *P* = 0.025), 1.26 (95% CI: 1.07–1.48, *P* = 0.006), and 1.29 (95% CI: 1.05–1.58, *P* = 0.015), respectively. Similarly, for PUFA, significant associations were identified in Q3 (OR: 1.18, 95% CI: 1.01–1.39, *P* = 0.036) and Q4 (OR: 1.26, 95% CI: 1.06–1.50, *P* = 0.009), while no significant association was observed for Q2 (*P* = 0.448).

### Subgroup analysis

**Fig. 4:**
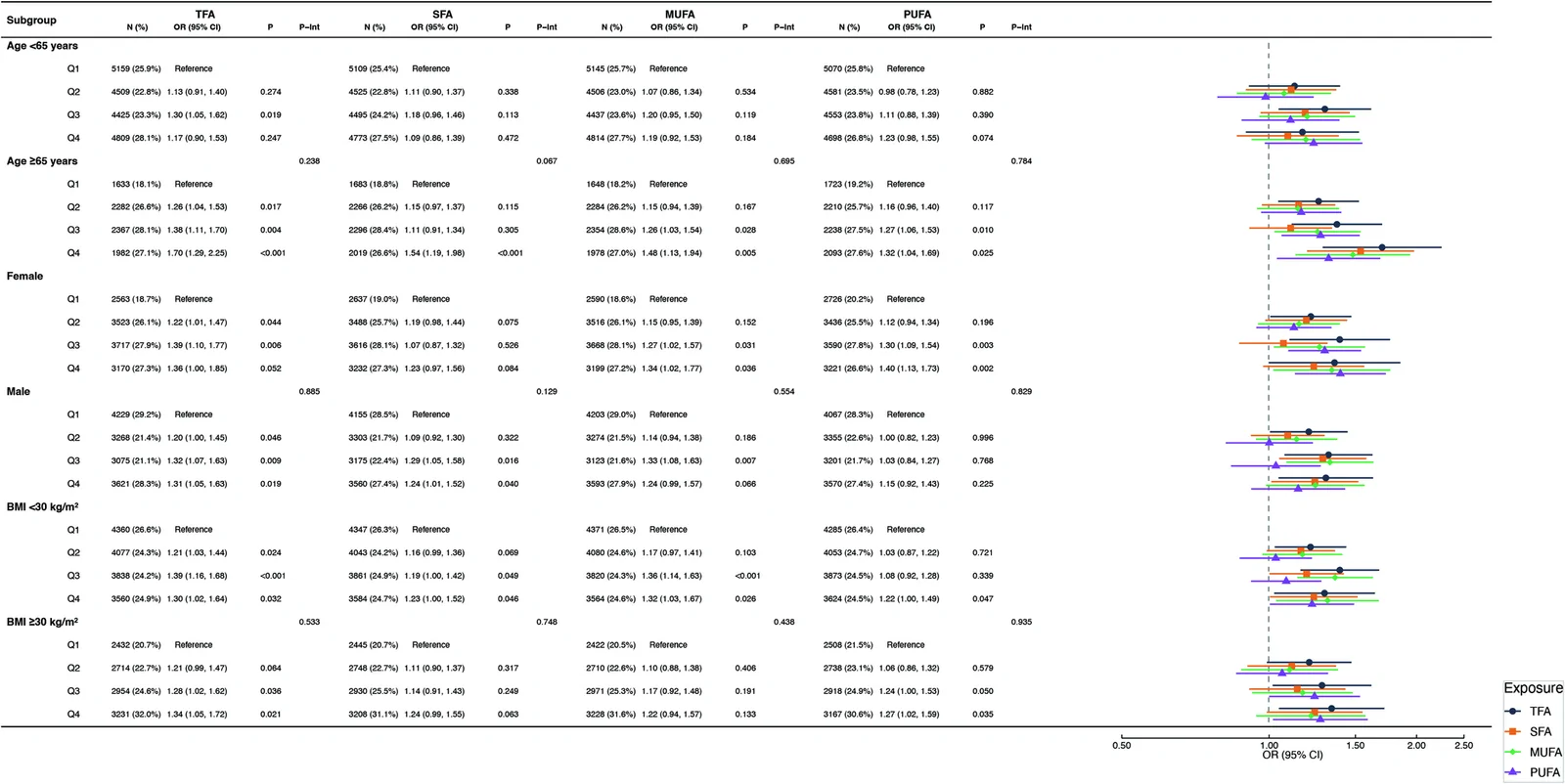
Subgroup analyses for associations between dietary fatty acid intake and advanced stage of cardiovascular-kidney-metabolic syndrome. ORs and 95% CIs were estimated using survey weighted logistic regression models adjusted for covariates shown in Model 2. Models were adjusted for age, sex, marital status, education level, race/ethnicity, poverty income ratio, physical activity, carbohydrate intake, protein intake, and total energy intake, except for the stratification variable within each subgroup. Q1 was used as the reference category. N (%) represents the unweighted sample size and weighted percentage. P for interaction was calculated from multiplicative interaction terms between fatty acid quartiles and subgroup variables. Advanced CKM was defined as stages 3–4 versus stages 1–2. BMI, body mass index; CI, confidence interval; TFA, total fatty acids; MUFA, monounsaturated fatty acids; OR, odds ratio; PUFA, polyunsaturated fatty acids; SFA, saturated fatty acids

Stratified analyses (Fig. 4) across different subgroups revealed that the links between dietary FA intake and the risk of advanced CKM stages were generally consistent across age (< 65 vs. ≥65 years), sex (male vs. female), race, and BMI (< 30 kg/m² vs. ≥30 kg/m²), none of the interaction terms reached statistical significance.

## Discussion

In this large, nationally representative sample of U.S. adults from NHANES 1999–2018, higher intakes of TFA, SFA, MUFA, and PUFA were independently associated with greater odds of advanced CKM stages. Dose–response analyses using restricted cubic splines further supported positive associations across all four FA categories, with largely linear relationships throughout the observed intake ranges. These associations remained robust in sensitivity analyses excluding participants with a history of cancer and were generally consistent across key subgroups defined by age, sex, and BMI. Nevertheless, because of the cross-sectional design, it remains possible that dietary behaviours were altered after the onset of CKM-related conditions, and the observed associations should therefore be interpreted cautiously.

Our findings suggest that not only the total amount, but also the quality and type of dietary FA intake may be associated with CKM severity and stage distribution. The observed positive associations with TFA, SFA, and MUFA highlight the need to revisit current dietary recommendations in the context of integrated cardiometabolic and renal risk. The observed positive associations across all major FA classes suggest that overall dietary fat exposure, dietary sources, and broader dietary patterns may be associated with advanced CKM stage distribution. Importantly, the biological effects of dietary FAs may differ substantially according to their food sources. For example, MUFA and PUFA derived from plant-based foods such as olive oil, nuts, seeds, and legumes may exert different metabolic effects from those derived from animal products or highly processed foods. Together, these results underscore the importance of dietary fat composition as a modifiable and potentially actionable factor in CKM prevention and management strategies. Notably, a recent NHANES-based study found that higher dietary omega-3 polyunsaturated fatty acid intake was associated with lower CKM stage severity and reduced mortality, further supporting the potential relevance of dietary fatty acids in CKM syndrome [29].

Biologically, several mechanisms may underlie these associations. Excess SFA intake can impair insulin signaling, promote hepatic and ectopic lipid accumulation, and exacerbate low-grade inflammation and oxidative stress, all key processes in the CKM pathway integrating metabolic dysfunction, renal injury, and cardiovascular risk [30, 31]. These mechanisms may contribute to the metabolic, renal, and cardiovascular abnormalities commonly observed among individuals with advanced CKM stages [32, 33]. While PUFA may counter some of these pathways via anti-inflammatory and lipid-modulating effects, net associations at the population level may be muted by variable sources and co-dietary patterns [34].

Some studies link higher total fat intake to adverse metabolic profiles and increased cardiovascular risk, while others emphasize the importance of the replacement nutrient and fat composition [30, 35]. Our findings suggest that the near-linear rise in advanced CKM odds with higher total fat intake likely reflects dietary clustering in U.S. settings, where higher-fat diets often coincide with greater consumption of SFA, animal-derived fats, ultra-processed foods, sodium, and refined carbohydrates [36].

The literature consistently shows that higher SFA intake negatively impacts lipid profiles, disrupts insulin signaling, and increases inflammatory and oxidative stress pathways [37]. Our results, showing a positive association between higher SFA quartiles and increased odds of advanced CKM. Biologically, SFA promotes hepatic and ectopic fat accumulation and the formation of lipotoxic intermediates, leading to endothelial dysfunction and glomerular injury [38, 39]. These mechanisms may contribute to the adverse metabolic, renal, and cardiovascular profiles that characterise advanced CKM stages [40].

Current research findings on MUFA are subject to differing interpretations. MUFA-rich patterns typified by olive oil and nuts (e.g., Mediterranean diets) often associate with better cardiometabolic outcomes [41]. However, in U.S. dietary contexts, a substantial share of MUFA derives from animal fats and mixed processed foods, frequently co-occurring with higher SFA, sodium, added sugars, and lower overall diet quality [42]. This distinction between plant-derived and animal-derived MUFA may be particularly important when interpreting observational findings, as these sources differ substantially in their accompanying nutrient profiles, food matrices, and overall dietary patterns. Therefore, associations observed for total MUFA intake should not be interpreted as reflecting the effects of any single food source, as the underlying food matrix may substantially modify biological responses and health outcomes. Our finding that the highest MUFA quartile (Q4) associates with ~ 12% higher odds of advanced CKM is consistent with analyses conducted within such U.S. environments and does not contradict evidence of benefit for plant-based, olive-oil–dominant MUFA patterns [43].

Many studies support PUFA, especially long-chain n–3 and appropriately balanced n–6, for lipid improvement, anti-inflammatory effects, and lower cardiovascular risk [44, 45]. However, evidence regarding their relationship with the integrated CKM syndrome remains limited. In the present study, higher PUFA intake was associated with increased odds of advanced CKM stages, particularly among participants in the upper intake quartiles. Several explanations may account for this finding. First, NHANES captures total PUFA intake without distinguishing specific subtypes, and potential benefits of marine-derived *n* − 3 FAs may be diluted by heterogeneous sources of *n* − 6 PUFA and plant-derived oils [46]. Moreover, PUFA from marine sources may have different biological effects from PUFA derived from vegetable oils or processed foods, owing to differences in fatty acid composition, accompanying nutrients, and dietary context. Second, higher PUFA intake may coexist with greater overall energy intake and broader dietary patterns that contribute to CKM progression. Third, the health effects of PUFA depend heavily on the nutrient being replaced and the overall dietary context. Individuals with higher PUFA intake may simultaneously consume greater amounts of total fat, energy, or ultra-processed foods, making it difficult to isolate the independent effect of PUFA itself. Therefore, the observed positive association between higher PUFA intake and advanced CKM stages should be interpreted with caution. Rather than contradicting the established cardiometabolic benefits of specific PUFA subtypes, this finding may reflect the heterogeneous nature of total PUFA intake captured in NHANES and the complex dietary contexts in which these FAs are consumed. Future studies incorporating detailed PUFA subtype profiling, dietary source information, and nutrient substitution analyses are needed to clarify the role of PUFA in CKM.

This study has important strengths, including the use of a large nationally representative NHANES sample with appropriate incorporation of sampling weights, strata, and primary sampling units, application of energy adjustment (residual method) for dietary exposures [19], comprehensive covariate control, dose–response modeling, and multiple robustness checks. From a clinical and public health perspective, our results underscore the potential importance of dietary fat quality in CKM prevention and management. While comprehensive dietary patterns remain central, reducing excessive intake of total and saturated fats and improving overall dietary fat quality may be relevant for individuals with or at risk of advanced CKM. Future studies should employ prospective designs to incorporate objective lipidomics, and distinguish specific FA subtypes and food matrices. Randomized feeding studies in CKM-enriched cohorts and pragmatic trials integrating dietitian-led interventions could clarify causality and translational impact.

Future studies should employ prospective cohort designs with repeated dietary assessments and incorporate objective lipidomic profiling to differentiate specific FA subtypes, metabolic intermediates, and their biological effects. In addition, food matrix interactions, preparation methods, and nutrient co-occurrence warrant further investigation. Integration of high-resolution metabolomics with gut microbiome data may also help uncover novel mechanistic pathways linking fat intake to CKM pathophysiology.

To enhance causal inference and translational relevance, randomized controlled trials in CKM-enriched or high-risk populations are needed, ideally testing isocaloric dietary fat modifications with long-term follow-up. Pragmatic trials that embed dietary interventions into routine care, led by dietitians or multidisciplinary teams—could help determine the feasibility and real-world effectiveness of fat-quality-focused strategies in slowing CKM progression. Ultimately, aligning nutritional epidemiology, mechanistic science, and implementation research will be key to advancing precision nutrition in CKM care. As CKM represents a unified multisystem syndrome rather than isolated organ dysfunctions, future research should prioritise integrated dietary strategies that address the shared metabolic roots and overlapping risk pathways across cardiovascular, kidney, and metabolic domains.

### Limitation

First, the cross-sectional design precludes causal inference and cannot exclude reverse causation (e.g., diet changes after disease recognition). Accordingly, the observed findings should be interpreted as associative rather than causal. Second, although the final analytical dataset contained no missing values, a proportion of participants were excluded during cohort construction because of incomplete information required for CKM staging, dietary assessment, or covariate adjustment. If excluded participants differed systematically from those included, selection bias may have been introduced, potentially affecting the representativeness of the study population and the generalisability of the findings. Third, 24-hour recalls are subject to recall and reporting biases and may not reflect habitual intake. Moreover, participants with advanced CKM may have modified their dietary habits because of underlying disease, medical advice, or disease-related symptoms (e.g., reduced appetite in heart failure or dietary restrictions associated with kidney disease), introducing the possibility of reverse causation. Consequently, the observed associations may not necessarily reflect the long-term dietary exposures that preceded CKM development. Fourth, despite adjustment for a broad range of demographic, socioeconomic, lifestyle, and dietary factors, residual confounding cannot be excluded. Important dietary characteristics such as sodium intake, food processing level, overall dietary quality, cooking methods, and specific food sources of FAs were not comprehensively captured and may partly explain the observed associations. In addition, unmeasured clinical factors, medication use, and healthcare-related behaviours may have influenced both dietary patterns and CKM status. Therefore, the observed associations should be interpreted cautiously. Fifth, CKM staging adapted to NHANES components may lead to some misclassification, and residual confounding from unmeasured factors (e.g., medication classes, dietary sodium, ultra-processed food intake) cannot be excluded. Finally, generalizability beyond U.S. adults and to clinical populations with distinct dietary patterns requires caution.

## Conclusion

This study demonstrated that higher dietary intakes of TFA, SFA, MUFA, and PUFA were associated with greater odds of advanced CKM stages. These findings highlight dietary fat intake as a potentially important correlate of CKM stage burden and support further prospective studies to clarify temporal and causal relationships. Future research should also evaluate whether modification of dietary fat quality and specific nutrient substitution strategies may contribute to CKM prevention and management.

## Supplementary Information

Below is the link to the electronic supplementary material.

Supplementary Material 1

## Data Availability

The study leverages data sourced from the National Health and Nutrition Examination Survey (NHANES) (https://www.cdc.gov/nchs/nhanes/index.htm.).

## Abbreviations

CKM: Cardiovascular-kidney-metabolic
eGFR: Estimated glomerular filtration rate
HDL-C: High-density lipoprotein cholesterol
MUFA: Monounsaturated fatty acids
PUFA: Polyunsaturated fatty acids
SFA: Saturated fatty acids
TFA: Total fatty acids
UACR: Urinary albumin to creatinine ratio
