# Supplementary material for: Dietary fatty acids intake and the risk of advanced cardiovascular-kidney-metabolic syndrome: evidence from NHANES 1999–2018
Source: Clin Exp Med. 2026 Jul 17;26(1):350. doi: 10.1007/s10238-026-02251-y (PMC13658389; doi:10.1007/s10238-026-02251-y)
Supplement: Supplementary file 1 [file 10238_2026_2251_MOESM1_ESM.docx]

Supplementary Table S1. Checklist for Strengthening the Reporting of Observational Studies in Epidemiology for this cohort study

Supplementary Table S2. Definitions of CKM

Supplementary Table S3. Detailed algorithm of the simplified 10-year cardiovascular disease risk models

Supplementary Table S4. Detailed algorithm for evaluating each CKM stage

Supplementary Table S5. Sensitivity analysis of fatty acid intake quartiles and advanced CKM syndrome without history of cancer

Supplementary Table S1. Checklist for Strengthening the Reporting of Observational Studies in Epidemiology for this cohort study

|  | Item No | Recommendation | Page No |
| --- | --- | --- | --- |
| **Title and abstract** | 1 | (*a*) Indicate the study’s design with a commonly used term in the title or the abstract | 1,3 |
|  |  | (*b*) Provide in the abstract an informative and balanced summary of what was done and what was found | 3 |
| Introduction | | | |
| Background/rationale | 2 | Explain the scientific background and rationale for the investigation being reported | 4 |
| Objectives | 3 | State specific objectives, including any prespecified hypotheses | 4 |
| Methods | | | |
| Study design | 4 | Present key elements of study design early in the paper | 5 |
| Setting | 5 | Describe the setting, locations, and relevant dates, including periods of recruitment, exposure, follow-up, and data collection | 5 |
| Participants | 6 | (*a*) Give the eligibility criteria, and the sources and methods of selection of participants. Describe methods of follow-up | 5 |
|  |  | (*b*) For matched studies, give matching criteria and number of exposed and unexposed | N/A |
| Variables | 7 | Clearly define all outcomes, exposures, predictors, potential confounders, and effect modifiers. Give diagnostic criteria, if applicable | 5,6 |
| Data sources/ measurement | 8* | For each variable of interest, give sources of data and details of methods of assessment (measurement). Describe comparability of assessment methods if there is more than one group | 5,6 |
| Bias | 9 | Describe any efforts to address potential sources of bias | 6 |
| Study size | 10 | Explain how the study size was arrived at | N/A |
| Quantitative variables | 11 | Explain how quantitative variables were handled in the analyses. If applicable, describe which groupings were chosen and why | 5,6 |
| Statistical methods | 12 | (*a*) Describe all statistical methods, including those used to control for confounding | 6 |
|  |  | (*b*) Describe any methods used to examine subgroups and interactions | 6 |
|  |  | (*c*) Explain how missing data were addressed | 6 |
|  |  | (*d*) If applicable, explain how loss to follow-up was addressed | N/A |
|  |  | (*e*) Describe any sensitivity analyses | 6 |
| Results | | |  |
| Participants | 13* | (a) Report numbers of individuals at each stage of study—eg numbers potentially eligible, examined for eligibility, confirmed eligible, included in the study, completing follow-up, and analysed | 6,7 |
|  |  | (b) Give reasons for non-participation at each stage | 6,7 |
|  |  | (c) Consider use of a flow diagram | Figure 1 |
| Descriptive data | 14* | (a) Give characteristics of study participants (eg demographic, clinical, social) and information on exposures and potential confounders | 6,7 |
|  |  | (b) Indicate number of participants with missing data for each variable of interest | Figure 1 |
|  |  | (c) Summarise follow-up time (eg, average and total amount) | N/A |
| Outcome data | 15* | Report numbers of outcome events or summary measures over time | 7 |
| Main results | 16 | (*a*) Give unadjusted estimates and, if applicable, confounder-adjusted estimates and their precision (eg, 95% confidence interval). Make clear which confounders were adjusted for and why they were included | 7 |
|  |  | (*b*) Report category boundaries when continuous variables were categorized | 7 |
|  |  | (*c*) If relevant, consider translating estimates of relative risk into absolute risk for a meaningful time period | N/A |
| Other analyses | 17 | Report other analyses done-eg analyses of subgroups and interactions, and sensitivity analyses | 7,8 |
| Discussion | | | |
| Key results | 18 | Summarise key results with reference to study objectives | 8 |
| Limitations | 19 | Discuss limitations of the study, taking into account sources of potential bias or imprecision. Discuss both direction and magnitude of any potential bias | 10 |
| Interpretation | 20 | Give a cautious overall interpretation of results considering objectives, limitations, multiplicity of analyses, results from similar studies, and other relevant evidence | 8,9,10 |
| Generalisability | 21 | Discuss the generalisability (external validity) of the study results | 9,10 |
| Other information | | | |
| Funding | 22 | Give the source of funding and the role of the funders for the present study and, if applicable, for the original study on which the present article is based | 2 |

*Give information separately for exposed and unexposed groups

**Note:** An Explanation and Elaboration article discusses each checklist item and gives methodological background and published examples of transparent reporting. The STROBE checklist is best used in conjunction with this article (freely available on the Web sites of PLoS Medicine at http://www.plosmedicine.org/, Annals of Internal Medicine at http://www.annals.org/, and Epidemiology at http://www.epidem.com/). Information on the STROBE Initiative is available at http://www.strobe-statement.org

Supplementary Table S2. Definitions of CKM

| **CKM conditions** | **Definition** | **CKM indicators** | **Threshold for CKM indicators** |
| --- | --- | --- | --- |
| CVD | Individuals with clinical CVD or subclinical CVD | Clinical CVD | History of chronic heart failure, coronary heart disease, heart attack, or stroke |
|  |  | Subclinical CVD | Any of the following criterion is met:  1) Very high-risk CKD in KDIGO classification: UACR ≥ 300 mg/g and eGFR ≤ 45-59 ml/min/1.73m2, UACR ≥ 30 mg/g and eGFR ≤ 30-44 ml/min/1.73m2, or eGFR ≤ 29 ml/min/1.73m2.  2) Predicted 10-year CVD risk ≥ 20% |
| Kidney diseases | Individuals with CKD | CKD | Moderate-to-high-risk CKD in KDIGO classification: UACR ≥ 30 mg/g and eGFR ≥ 60 ml/min/1.73m2, UACR < 300 mg/g and eGFR ≤ 45-59 ml/min/1.73m2, or UACR < 30 mg/g and eGFR ≤ 30-44 ml/min/1.73m2. |
| Metabolic disorders | Individuals with overweight/obesity, abdominal obesity, prediabetes, diabetes, hypertension, hypertriglyceridemia or MetS | Overweight/obesity | BMI ≥25 kg/m2 (or ≥23 kg/m2 if Asian ancestry) * |
|  |  | Abdominal obesity | Waist circumference ≥88/102 cm in female/male (or if Asian ancestry ≥80/90 cm in female/male) |
|  |  | Prediabetes | Fasting blood glucose ≥ 100-124 mg/dL or HbA1c ≥ 5.7%-6.4% and without self-reported diagnosis of diabetes, use of insulin, or oral hypoglycemic agents |
|  |  | Diabetes | Fasting blood glucose ≥ 125 mg/dL or HbA1c ≥ 6.5% or self-reported diagnosis of diabetes, use of insulin, or oral hypoglycemic agents |
|  |  | Hypertension | SBP ≥130 mm Hg or DBP ≥80 mm Hg or self-reported diagnosis of hypertension or use of antihypertensive medications |
|  |  | Hypertriglyceridemia | Triglycerides ≥ 135 mg/dL |
|  |  | MetS | MetS is defined by the presence of 3 or more of the following: 1) Waist circumference ≥88/102 cm in female/male (or if Asian ancestry ≥80/90 cm in female/male). 2) HDL cholesterol <50/40 mg/dL in female/male. 3) Triglycerides ≥150 mg/dL. 4) Elevated blood pressure (SBP ≥130 mm Hg or DBP ≥80 mm Hg and/or use of antihypertensive medications) 5) Fasting blood glucose ≥100 mg/dL |

*Asian was not listed as a separate race/ethnicity until NAHNES 2011-2012, therefore the uniform threshold for BMI and waist circumference was used in all participants in NHANES 1999-2010.

Abbreviations: BMI = body mass index; CKD = chronic kidney disease; CKM = cardiovascular-kidney-metabolic syndrom; CVD = cardiovascular disease; DBP = diastolic blood pressure; eGFR = estimated glomerular filtration rate; HDL-C = high-density lipoprotein cholesterol; KDIGO = The Kidney Disease: Improving Global Outcomes; MetS = metabolic syndrome; SBP = systolic blood pressure; UACR = urinary albumin to creatinine ratio.

Supplementary Table S3. Detailed algorithm of the simplified 10-year cardiovascular disease risk models

| **Sex** | **Calculation** |
| --- | --- |
| Women | log-Odds = -3.307728 + 0.7939329 × (age – 55) /10 + 0.0305239 × (TC – HDL-C – 3.5) – 0.1606857 × (HDL-C – 1.3) /0.3 – 0.2394003 × (min(SBP, 110) – 110) /20 + 0.360078 × (max(SBP, 110) – 130) /20 + 0.8667604 × (if diabetes) + 0.5360739 × (if current smoker) + 0.6045917 × (min(eGFR, 60) – 60) / -15 + 0.0433769 × (max(eGFR, 60) – 90) / -15 + 0.3151672 × (if using anti-hypertensive medication) – 0.1477655 × (if using statin) – 0.0663612 × (if using anti-hypertensive medication) × (max(SBP, 110) – 130) /20 + 0.1197879 × (if using statin) × (TC – HDL-C – 3.5) – 0.0819715 × (age – 55) /10 × (TC – HDL-C – 3.5) + 0.0306769 × (age – 55) /10 × (HDL-C – 1.3) /0.3 – 0.0946348 × (age – 55) /10 × (max(SBP, 110) – 130) /20 – 0.27057 × (age – 55) /10 × (if diabetes) – 0.078715 × (age – 55) /10 × (if current smoker) – 0.1637806 × (age – 55) /10 × (min(eGFR, 60) – 60) / -15  Risk = exp(log-Odds) / (1 + exp(log-Odds)) |
| Men | log-Odds = -3.031168 + 0.7688528 × (age – 55) /10 + 0.0736174 × (TC – HDL-C – 3.5) – 0.0954431 × (HDL-C – 1.3) /0.3 – 0.4347345 × (min(SBP, 110) – 110) /20 + 0.3362658 × (max(SBP, 110) – 130) /20 + 0.7692857 × (if diabetes) + 0.4386871 × (if current smoker) + 0.5378979 × (min(eGFR, 60) – 60) / -15 + 0.0164827 × (max(eGFR, 60) – 90) / -15 + 0.288879 × (if using anti-hypertensive medication) – 0.1337349 × (if using statin) – 0.0475924 × (if using anti-hypertensive medication) × (max(SBP, 110) – 130) /20 + 0.150273 × (if using statin) × (TC – HDL-C – 3.5) – 0.0517874 × (age – 55) /10 × (TC – HDL-C – 3.5) + 0.0191169 × (age – 55) /10 × (HDL-C – 1.3) /0.3 – 0.1049477 × (age – 55) /10 × (max(SBP, 110) – 130) /20 – 0.2251948 × (age – 55) /10 × (if diabetes) – 0.0895067 × (age – 55) /10 × (if current smoker) – 0.1543702 × (age – 55) /10 × (min(eGFR, 60) – 60) / -15  Risk = exp(log-Odds) / (1 + exp(log-Odds)) |

Abbreviations: eGFR = estimated glomerular filtration rate; HDL-C = high-density lipoprotein cholesterol; SBP = systolic blood pressure; TC = total cholesterol.

Supplementary Table S4. Detailed algorithm for evaluating each CKM stage

| **CKM stages** | **Definition** | **Criterion** | **Threshold for CKM conditions** |
| --- | --- | --- | --- |
| Stage 0: No CKM risk factors | Individuals with normal BMI and waist circumference, normoglycemia, normotension, a normal lipid profile, and no evidence of CKD or subclinical or clinical CVD | All criteria are met | BMI <25 kg/m^2^ (or <23 kg/m^2^ if Asian ancestry)^*^ |
|  |  |  | Waist circumference <88/102 cm in female/male (or if Asian ancestry <80/90 cm in female/male) |
|  |  |  | Fasting blood glucose < 100 mg/dL and HbA1c < 5.7% and without self-reported diagnosis of diabetes, use of insulin, or oral hypoglycemic agents |
|  |  |  | SBP <130 mm Hg and DBP <80 mm Hg without self-reported diagnosis of hypertension or use of antihypertensive medications |
|  |  |  | HDL cholesterol >50/40 mg/dL in female/male and triglycerides < 150 mg/dL |
|  |  |  | Low-risk CKD in KDIGO classification according to eGFR and UACR: UACR < 30 mg/g and eGFR ≥ 60 ml/min/1.73m^2^. |
|  |  |  | Predicted 10-year CVD risk < 20% |
|  |  |  | No clinical CVD |
| Stage 1: Excess or dysfunctional adiposity | Individuals with overweight/obesity, abdominal obesity, or dysfunctional adipose tissue, without the presence of other metabolic risk factors or CKD | Any of the three criteria is met | Overweight/obesity |
|  |  |  | Abdominal obesity |
|  |  |  | Prediabetes |
|  |  | All criteria are met | SBP <130 mm Hg and DBP <80 mm Hg without self-reported diagnosis of hypertension or use of antihypertensive medications |
|  |  |  | HDL cholesterol >50/40 mg/dL in female/male and triglycerides <150 mg/dL |
|  |  |  | Low-risk CKD in KDIGO classification according to eGFR and UACR: UACR < 30 mg/g and eGFR ≥ 60 ml/min/1.73m^2^ |
|  |  |  | Predicted 10-year CVD risk < 20% |
|  |  |  | No clinical CVD |
| Stage 2: Metabolic risk factors and CKD | Individuals with metabolic risk factors (hypertriglyceridemia, hypertension, MetS, diabetes), or CKD | Any of the five criteria is met | Hypertriglyceridemia |
|  |  |  | Hypertension |
|  |  |  | diabetes |
|  |  |  | MetS |
|  |  |  | Moderate-to-high-risk CKD in KDIGO classification |
|  |  | All criteria are met | No very high-risk CKD in KDIGO classification |
|  |  |  | Predicted 10-year CVD risk < 20% |
|  |  |  | No clinical CVD |
| Stage 3: Subclinical CVD in CKM | Subclinical CVD among individuals with excess/dysfunctional adiposity, other metabolic risk factors, or CKD | Any of the two criteria is met | Very high-risk CKD in KDIGO classification |
|  |  |  | Predicted 10-year CVD risk ≥ 20% |
|  |  | Any of the eight criteria is met | Overweight/obesity |
|  |  |  | Abdominal obesity |
|  |  |  | Prediabetes |
|  |  |  | Hypertriglyceridemia |
|  |  |  | Hypertension |
|  |  |  | diabetes |
|  |  |  | MetS |
|  |  |  | Moderate-to-high-risk CKD in KDIGO classification |
|  |  | The criterion is met | No clinical CVD |
| Stage 4: Clinical CVD in CKM | Clinical CVD among individuals with excess/dysfunctional adiposity, other metabolic risk factors, or CKD | The criterion is met | Clinical CVD |
|  |  | Any of the nine criteria is met | Overweight/obesity |
|  |  |  | Abdominal obesity |
|  |  |  | Prediabetes |
|  |  |  | Hypertriglyceridemia |
|  |  |  | Hypertension |
|  |  |  | diabetes |
|  |  |  | MetS |
|  |  |  | Moderate-to-high-risk CKD in KDIGO classification |
|  |  |  | Very high-risk CKD in KDIGO classification |

*Asian was not listed as a separate race/ethnicity until NHANES 2011-2012, therefore the uniform threshold for BMI and waist circumference was used in all participants in NHANES 1999-2010.

Abbreviations: BMI = body mass index; CKD = chronic kidney disease; CKM = cardiovascular-kidney-metabolic syndrom; CVD = cardiovascular disease; DBP = diastolic blood pressure; eGFR = estimated glomerular filtration rate; HDL-C = high-density lipoprotein; KDIGO = The Kidney Disease: Improving Global Outcomes; NHANES = National Health and Nutrition Examination Survey; SBP = systolic blood pressure; UACR = urinary albumin to creatinine ratio.

Supplementary Table S5. Sensitivity analysis of fatty acid intake quartiles and advanced CKM syndrome without history of cancer

|  | **Model 1** | | **Model 2** | | |
| --- | --- | --- | --- | --- | --- |
|  | **OR (95% CI)** | ***P*** | | **OR (95% CI)** | ***P*** |
| **TFA quartiles** | |  | |  |  |
| Q1 | *Reference* |  | | *Reference* |  |
| Q2 | 1.47 (1.30, 1.65) | < 0.001 | | 1.20 (1.04, 1.38) | 0.01 |
| Q3 | 1.53 (1.36, 1.71) | < 0.001 | | 1.33 (1.14, 1.54) | < 0.001 |
| Q4 | 1.17 (1.02, 1.34) | 0.021 | | 1.28 (1.05, 1.56) | 0.016 |
| **SFA quartiles** | |  | |  |  |
| Q1 | *Reference* |  | | *Reference* |  |
| Q2 | 1.38 (1.22, 1.55) | < 0.001 | | 1.13 (0.99, 1.30) | 0.076 |
| Q3 | 1.38 (1.23, 1.55) | < 0.001 | | 1.20 (1.03, 1.40) | 0.021 |
| Q4 | 1.13 (1.00, 1.28) | 0.058 | | 1.20 (1.01, 1.44) | 0.039 |
| **MUFA quartiles** |  |  | |  |  |
| Q1 | Reference |  | | Reference |  |
| Q2 | 1.42 (1.25, 1.62) | < 0.001 | | 1.19 (1.02, 1.40) | 0.025 |
| Q3 | 1.43 (1.27, 1.61) | < 0.001 | | 1.26 (1.07, 1.48) | 0.006 |
| Q4 | 1.19 (1.04, 1.35) | 0.01 | | 1.29 (1.05, 1.58) | 0.015 |
| **PUFA quartiles** |  |  | |  |  |
| Q1 | Reference |  | | Reference |  |
| Q2 | 1.25 (1.10, 1.42) | < 0.001 | | 1.06 (0.91, 1.23) | 0.448 |
| Q3 | 1.40 (1.24, 1.59) | < 0.001 | | 1.18 (1.01, 1.39) | 0.036 |
| Q4 | 1.20 (1.06, 1.36) | 0.004 | | 1.26 (1.06, 1.50) | 0.009 |

Model 1: Unadjusted;

Model 2: Adjusted for age, sex, marital status, education level, race/ethnicity, poverty income ratio, physical activity, carbohydrate intake, protein intake, and total energy intake.

ORs and 95% CIs were estimated using survey weighted logistic regression models accounting for NHANES sampling weights, strata, and primary sampling units. Quartile 1 (Q1) served as the reference category. As a sensitivity analysis, participants with a history of cancer were excluded.

Abbreviations: SFA, saturated fatty acids; TFA, total fatty acids; MUFA, monounsaturated fatty acids; PUFA, polyunsaturated fatty acids; OR, odds ratio; CI, confidence interval.
